# DNA/RNA Preservation in Glacial Snow and Ice Samples

**DOI:** 10.3389/fmicb.2022.894893

**Published:** 2022-05-23

**Authors:** Christopher B. Trivedi, Christoph Keuschnig, Catherine Larose, Daniel Vasconcelos Rissi, Rey Mourot, James A. Bradley, Matthias Winkel, Liane G. Benning

**Affiliations:** ^1^Interface Geochemistry, GFZ German Research Centre for Geosciences, Potsdam, Germany; ^2^Environmental Microbial Genomics, Université de Lyon, Ecully Cedex, France; ^3^Department of Earth Sciences, Freie Universität Berlin, Berlin, Germany; ^4^School of Geography, Queen Mary University of London, London, United Kingdom

**Keywords:** DNA/RNA preservation, glacial microbiology, metagenomics, metatranscriptomics, RNAlater, Zymo DNA/RNA Shield, flash freezing

## Abstract

The preservation of nucleic acids for high-throughput sequencing is an ongoing challenge for field scientists. In particular, samples that are low biomass, or that have to be collected and preserved in logistically challenging environments (such as remote sites or during long sampling campaigns) can pose exceptional difficulties. With this work, we compare and assess the effectiveness of three preservation methods for DNA and RNA extracted from microbial communities of glacial snow and ice samples. Snow and ice samples were melted and filtered upon collection in Iceland, and filters were preserved using: (i) liquid nitrogen flash freezing, (ii) storage in RNAlater, or (iii) storage in Zymo DNA/RNA Shield. Comparative statistics covering nucleic acid recovery, sequencing library preparation, genome assembly, and taxonomic diversity were used to determine best practices for the preservation of DNA and RNA samples from these environments. Our results reveal that microbial community composition based on DNA was comparable at the class level across preservation types. Based on extracted RNA, the taxonomic composition of the active community was primarily driven by the filtered sample volume (i.e., biomass content). In low biomass samples (where <200 ml of sample volume was filtered) the taxonomic and functional signatures trend toward the composition of the control samples, while in samples where a larger volume (more biomass) was filtered our data showed comparable results independent of preservation type. Based on all comparisons our data suggests that flash freezing of filters containing low biomass is the preferred method for preserving DNA and RNA (notwithstanding the difficulties of accessing liquid nitrogen in remote glacial field sites). Generally, RNAlater and Zymo DNA/RNA Shield solutions work comparably well, especially for DNA from high biomass samples, but Zymo DNA/RNA Shield is favored due to its higher yield of preserved RNA. Biomass quantity from snow and ice samples appears to be the most important factor in regards to the collection and preservation of samples from glacial environments.

## Introduction

The preservation of biological materials for generating genomic data is essential to describe the diversity, function, and regulation of life and associated environmental processes in various habitats. When the studied habitats are remote and/or logistically challenging (e.g., polar, deep sea, etc.) effective preservation of short-lived nucleic acids, such as RNA, is critical prior to downstream analysis. The objective of nucleic acid preservation from environmental samples is to simultaneously retain the highest possible yield of nucleic acid from a sample, while also obtaining a representative signature of the biological composition, activity, and function of the environment at the time of observation and sampling. Specifically, preservation should (i) minimize nucleic acid degradation, and in the case of RNA, (ii) minimize further or differential expression of genes after sampling, and (iii) minimize non-random expression or degradation of nucleic acids during and following sampling and transit. Here we evaluate sample preservation efficacy on nucleic acid yield from glacial snow and ice samples.

At room temperatures, RNA can be quickly degraded *via* ribonuclease (RNAse) activity. Some estimates from yeast growth show the decay of messenger RNA (mRNA) at an average half-life of 4.8 min (Chan et al., 2018). Nucleic acid preservation methods are more commonly developed, tested, and validated for the handling of human and culture tissues in life science research, rather than in natural environments and ecosystems. Tested preservation methods for human tissue samples include flash freezing (typically by immersing samples in liquid nitrogen and subsequent storage at −80°C; Seelenfreund et al., 2014), formalin-fixed and paraffin-embedding (FFPE; Wimmer et al., 2018), or storage in commercial chemical preservation buffers (e.g., RNAlater; Salehi and Najafi, 2014; Martin et al., 2017). Most often for medical tissue samples, flash freezing remains the favored and most optimal preservation method (e.g., when compared to FFPE, Medeiros et al., 2007). However, flash freezing requires access to liquid nitrogen, which for ecosystem research during long sampling campaigns and/or in a remote field setting such as glacial environments, may be challenging. For studies on samples from human tissues (Mutter et al., 2004; Wang et al., 2006) and wildlife gastrointestinal microbiomes (Menke et al., 2017), comparisons of flash freezing versus chemical buffers have generally found that the use of commercial buffers (as well as lab-made salt buffers) can be as effective as flash freezing for RNA preservation. Furthermore, Bundgaard-Nielsen et al. (2018) compared flash freezing, RNAlater, and Zymo DNA/RNA Shield, to determine the best method for the preservation of human fecal samples and found all methods to be comparable when considering bacterial composition using 16S rRNA gene sequencing. However, these studies focus on the preservation of bacteria or human tissues and not environmental micro-eukaryotes.

Alternatively, chemical preservatives like RNAlater and Zymo DNA/RNA Shield have been used widely in field studies due to their ease of transport and handling (typically at room temperature). RNAlater has been effectively used to preserve environmental samples including freshwater bacterial communities (McCarthy et al., 2015), deep-sea sediments (Zhang et al., 2016), and arsenic-rich river sediments (Herrera et al., 2021). Zymo DNA/RNA Shield solution has been used successfully to preserve tongue epithelial samples from cattle (Horsington et al., 2020), and showed similar preservation efficacy for bird fecal microbiome samples when compared with flash freezing (Knutie and Gotanda, 2018). Additionally, comparing Zymo DNA/RNA Shield and RNAlater when investigating DNA preservation of rainbow trout gut contents (Hildonen et al., 2019), and RNA from human blood samples (Soliman, 2018) showed them to be comparable. RNAlater has also been used in glacial ecosystems for the preservation of bacterial RNA in subglacial sediments (Hamilton et al., 2013), cryoconites (Segawa et al., 2020), and proglacial meltwaters (Sheik et al., 2015), while Zymo DNA/RNA Shield has been used to characterize the bacterial communities of a sulfur-rich glacial ecosystem (Trivedi et al., 2018, 2020). However, most of these were comparative studies focused on bacterial RNA preservation and, to date, no study has been conducted which focus specifically on the efficacy of preservation types of bacteria and micro-eukaryote biomass from polar snow and ice ecosystems.

To fill this gap, we compared the preservation potential of DNA and RNA in pigmented eukaryotic algae-rich snow and ice samples from Iceland glaciers using: flash freezing in liquid nitrogen, RNAlater, and Zymo DNA/RNA Shield (hereafter referred to as Freeze or F, RNAlater or R, and Zymo or Z, respectively). For each method, we compared statistics from nucleic acid recovery, sequencing library preparation, genome assembly, and taxonomic diversity and relatedness. Additionally, because the biomass in our samples is dominated by eukaryotic algal communities, we have, for the RNA, also evaluated multiple library preparations including totalRNA, ribodepleted RNA, and poly(A) selected RNA. We show that total and active microbial community composition was comparable at the class level across preservation types. We recommend flash freezing for preservation of glacial DNA and RNA in microbial dominated snow and ice samples, but note that chemical preservation can work comparably well.

## Materials and Methods

### Sample Types, Collection, and Preservation Methods

**FIGURE 1:**
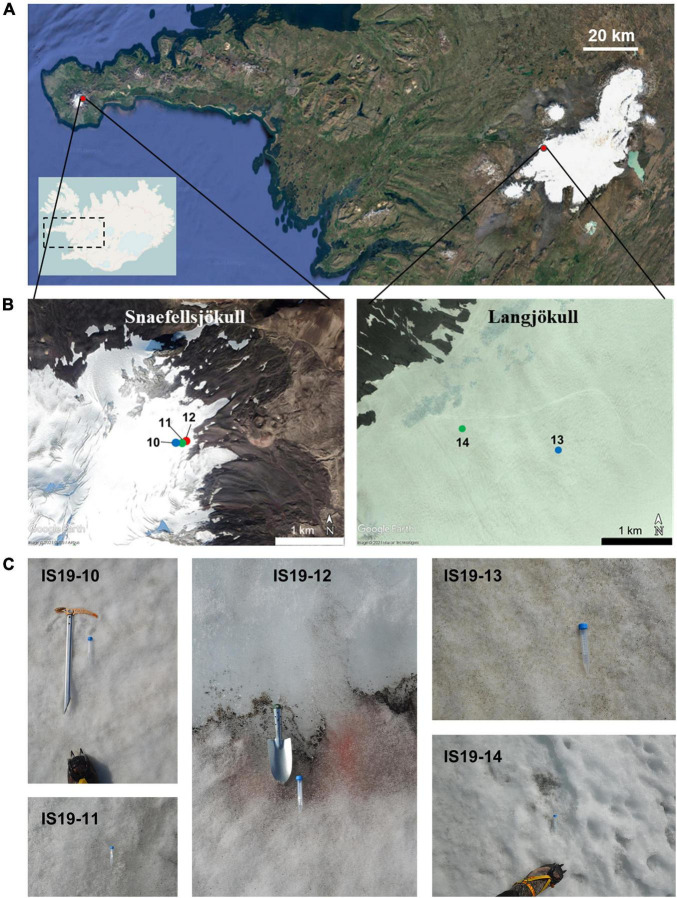
Satellite images showing the location of sampled Icelandic glaciers and close-up pictures of sample sites. **(A)** The top satellite image is zoomed in from the inset line map of Iceland. Glacier locations are marked with red dots with further zoomed-in images highlighting individual glaciers and site numbers. Blue dots indicate snow sites, green dots indicate ice sites, and the red dot indicates a snow-ice interface. **(B)** Sites IS19-10, IS19-11, and IS19-12 were located on Snaefellsjökull (B-left; scale: 1 km) and sites IS19-13 and IS19-14 on Langjökull (B-right; scale: 1 km) Further location and site details can be found in Table 1. **(C)** Sites are arranged from left to right by sampling location: Snaefellsjökull (IS19-10, −11, and −12) and Langjökull (IS19-13 and −14). Note the differences in sample types: snow (IS19-10 and −13), ice (IS19-11 and −14), and snow-ice interface (IS19-12). Inset line map courtesy of OpenStreetMap and satellite images courtesy of Google Earth.

Snow and ice samples were collected in August 2019 from two glaciers in Iceland (Snaefellsjökull and Langjökull, respectively, see Figure 1) following previously established protocols (Lutz et al., 2015a; Winkel et al., 2022). For this study, we specifically targeted samples that contained high quantities of visible mineral and microbial particles [equivalent to “dirty snow” and “dirty ice” habitats described in Lutz et al. (2014)]. We collected samples from two snow habitats (sites IS19-10 and IS19-13), two ice habitats (sites IS19-11 and IS19-14) and one interface between microbially rich snow and ice habitats (site IS19-12). On both glaciers, the snow samples were collected at a higher elevation than the ice samples (Figure 1 and Table 1). All samples were collected into sterile whirl-pak (Kleinfeld Labortechnik GmbH, Gehrden, Germany) bags using ethanol-sterilized plastic (snow) or stainless-steel (ice) shovels. Upon return to the field lab and within a maximum of ∼ 6 h, samples were thawed (at ∼10–15°C) for processing.

**TABLE 1:** Location and sample information.

Glacier	Site name	Field sample type	Coordinates	Elevation (m)
Snaefellsjökull	IS19-10	Snow	64.81406° N 23.75144° W	947
Snaefellsjökull	IS19-11	Ice	64.81413° N 23.75032° W	940
Snaefellsjökull	IS19-12	Snow/ice interface	64.81451° N 23.74922° W	919
Langjökull	IS19-13	Dirty snow	64.68199° N 20.50778° W	1145
Langjökull	IS19-14	Dark ice	64.63456° N 20.53851° W	961

*Info includes the name of the glaciers where samples were collected, the site name, the field sample type, site coordinates, and elevation.*

For total organic carbon (TOC), samples were filtered (between 0.5 and 1 L) through 0.7 μm ashed GFF filters. The solids were freeze-dried and ball-milled (MM2000; Retsch GmbH, Haan, Germany) to a fine powder. The milled material was analyzed using a Carlo Erba NC-2500 elemental analyzer. Reproducibility was better than 0.1 wt% C based on repeatedly measured standards.

For DNA/RNA, between 100 and 500 mL of each sample was filtered (Table 2) through a single-use sterile filter unit containing a removable 47 mm diameter cellulose nitrate filter (0.2 μm pore size) (Thermo Fisher Scientific, Henningsdorf, Germany). Multiple replicate aliquots were processed from each sample to test the effects of three DNA and RNA preservation methods on downstream processed metagenomes and metatranscriptomes. Individual filters for each sample were preserved in three ways: (1) *Freeze*: immediately frozen in a cryo-shipper (CX-100, Worthington Industries, Burscheid, Germany) already primed with liquid nitrogen, (2) *RNAlater*: preserved in RNAlater^®^ (Thermo Fisher Scientific), and (3) *Zymo*: inserted into a ZymoBIOMICS lysis tube containing 750 μl of DNA/RNA Shield*™* (Zymo Research Corp., Freiburg, Germany). Hereafter, for simplicity, we list the samples with a sample short name (Table 2) that contains the sample site number followed by an F, L, or Z to represent the three tested preservation methods (*Freeze*, *RNAlater*, and *Zymo*). Both the *RNAlater-* and *Zymo-* preserved filters were kept at 4°C until returning to the home institution, followed by storage at −20°C until DNA/RNA extraction. The *Freeze* filters were, upon return to the home laboratory, transferred into a −80°C freezer where they were stored until DNA and RNA extraction. A field blank, which consisted of autoclaved ultrapure water was subjected to the same filtration process as described above in the field laboratory and the resulting filter was preserved in a ZymoBIOMICS lysis tube and processed downstream following the same protocol as all other field samples.

**TABLE 2:** Quality control statistics for the various tested sample preservation methods and additional sample data.

					Extraction conc. per 100 ml filtered (ng/μl)		Library conc (nM)				Seq yield (Mbp)			
Site name and habitat	Preservation type	Sample short name	Filtered volume (ml)	TOC (wt. %)	DNA	RNA	DNA	TotalRNA	Ribodepleted	Poly(A) selected	DNA	TotalRNA	Ribodepleted	Poly(A) selected
IS19-10 Snow	Freeze	10F	500	2.92	39.8	BDL	5.87	699.2	112.3	−	267	402	358	−
	RNA later	10L	250		16.7	BDL	31.89	46.6	−	−	361	188	−	−
	Zymo	10Z	250		11	BDL	11.34	28.2	0.1	−	365	210	95	−
IS19-11 Ice	Freeze	11F	320	4.08	21	BDL	4.41	163.3	14.6	−	155	328	321	−
	RNA later	11L	250		1.8	BDL	6.56	34	−	−	153	173	−	−
	Zymo	11Z	250		6.2	BDL	38.34	13.3	2	−	325	131	167	−
IS19-12 Snow/ice interface	Freeze	12F	250	6.8	192	0.21	2.39	343.7	230	10.9	249	2225	792	372
	RNA later	12L	100		97.9	0.6	2.46	692.5	252	18.2	339	691	534	229
	Zymo	12Z	100		590	0.8	42.19	515.7	199.5	98.4	183	851	612	267
IS19-13 Snow	Freeze	13F	250	0.33	14.4	BDL	2.69	78.8	13.4	−	269	252	181	−
	RNA later	13L	100		2.8	BDL	BDL	18.1	−	−	2	102	−	−
	Zymo	13Z	150		27.2	BDL	43.11	63.6	2.1	−	361	274	165	−
IS19-14 Ice	Freeze	14F	300	0.28	12.6	BDL	1.13	24.5	1.5	−	296	36	278	−
	RNA later	14L	100		2.2	BDL	BDL	0.8	−	−	<1	68	−	−
	Zymo	14Z	100		27.4	BDL	15.84	1.4	0.3	−	369	100	34	−
Field Blank	Zymo	FieldBL	100	NA	0.7	BDL	BDL	0.8	0.4	0.4	14	57	55	34
Extraction Blank	−	LabBL	−		BDL	BDL	0.1	8	−	−	2	78	−	−
NEG	−	NEG	−		−	−	−	0.7	0.5	2.6	−	3	208	139

*Samples are organized by site and with each preservation type and associated short name.*

*Data for filtered volume and TOC content of each sample (Winkel et al., 2022) are shown.*

*The DNA and RNA concentrations are normalized per 100 ml sample volume filtered and reveal that the RNA concentrations were BDL in all but the highest biomass (TOC) IS19-12 sample. Shown also are the library concentration as determined by qPCR, and total sequencing yield from the paired end 2 × 250 bp sequencing run.*

*Values with a hyphen (-) refer to samples that were not prepared or processed for that preservation type.*

*BDL, below detection limit of the Qubit RNA HS kit (0.25 ng/μl), or below detection limit of qPCR library standards (0.2 pM).*

*Mbp, 10^6^ basepairs (LOD is 1,000 bp).*

### DNA/RNA Extraction and Metagenomic Library Preparation

All DNA and RNA extractions were performed in a sterile laminar flow hood and all samples including the field blank (FieldBL), and a laboratory extraction blank (LabBL; laboratory-grade water) were extracted using the ZymoBIOMICS DNA/RNA Mini Kit (Zymo Research Corp.). All *Zymo*- and *Freeze*-preserved samples were extracted following standard procedures, while the *RNAlater*-preserved samples required additional handling steps prior to them being usable in the ZymoBIOMICS workflow due to the high salt content of the RNAlater solution. This comprised of gently rinsing each filter multiple times (at least three) with a 10% PBS solution until all salt crystals were dissolved (Saito et al., 2011). Extractions were performed according to the manufacturer’s protocol and DNA and RNA from all treatments were finally eluted in 50 μl. Extracted DNA concentrations were determined using a Qubit 2.0 fluorometer (Thermo Fisher Scientific) and the Qubit dsDNA HS assay (Thermo Fisher Scientific). Extracted DNA and RNA concentrations were normalized to an equal sample volume and are reported per 100 ml filtered (Table 2). Prior to library preparation, DNA samples were diluted to 0.2 ng/μl as recommended by the library preparation kit, while RNA samples were used directly for library preparation as most were measured as below detection (BDL).

Metagenomic libraries were prepared using the Nextera XT DNA Library Preparation Kit (Illumina, Évry, France) in conjunction with the Nextera XT index kit v2 (Illumina, Évry, France) following the reference guide (Document # 15031942 v03) provided by Illumina. Concentrations of libraries from each sample were measured by quantitative PCR (qPCR) (Corbett Rotor-Gene 6000 real-time PCR cycler) using primers targeting the p5 and p7 adapters (ProNex^®^ NGS Library Quant Kit, Promega Corp., Charbonnières-les-Bains, France) to ensure measurement of only intact libraries, which would result in a read. All library preparations were quality checked *via* qPCR and on an Agilent 2100 Bioanalyzer (Agilent Genomics, Les Ulis, France) either with a DNA 1000 chip or a DNA High Sensitivity chip to ensure the absence of primer dimers and estimate the size distribution of DNA fragments in each library. Molarities of each sample were calculated based on the mean length of DNA fragments and the concentrations measured by qPCR (Table 2). Subsequently, an equimolar pool was prepared from all libraries which was again quality checked by running a Bioanalyzer chip (Supplementary Figure 1A). The final pool was loaded on a V2-flow cell for a 2 × 250 paired-end sequencing run on an Illumina MiSeq according to the manufacturer’s instructions.

### cDNA Library Preparation for TotalRNA, Ribodepleted, and Poly(A) Selected RNA

Three different cDNA libraries were prepared in order to compare RNA extraction efficiencies for preservation type and to test for sequencing differences between totalRNA, ribodepletion, and poly(A) selection library preparation (Table 2). This was done because ribodepletion ideally captures a better bacterial signal while poly(A) selection is better for the eukaryotic signal. DNA and totalRNA libraries were prepared for all samples. Ribodepleted libraries were created for *Freeze*-preserved and *Zymo*-preserved samples of all sites, but not for *RNAlater*-preserved samples (except for site IS19-12 due to its uniqueness and high biomass; TOC–6.8%, Table 2). Additionally, for site IS19-12 poly(A) selected libraries was prepared for each preservation type. The field blank (FieldBL) was prepared for all preservation types, while an extraction blank (LabBL) was prepared for DNA and totalRNA libraries. Additionally, a negative control (nuclease-free water; NEG in Table 2) was prepared for all three RNA libraries (totalRNA, ribodepleted, and poly(A) selected).

TotalRNA libraries were prepared using the NEBNext Ultra II Direction RNA Library Prep kit for Illumina (New England Biolabs, Évry-Courcouronnes, France). Extracted RNA concentrations were below the detection limit for almost all samples (except sample IS19-12) and therefore was directly used in library preparation after extraction rather than being normalized to a standard concentration. Preparation of the ribodepleted libraries was carried out using the above kit but with the addition of the NEBNext rRNA Depletion Kit module (New England Biolabs). The manufacturer’s protocol was followed exactly using 12 μL of RNA directly (no dilution). The RNA was fragmented for 8 min and 16 cycles were used for the library amplification. Similarly, preparation of the poly(A) selected libraries was carried out according to the manufacturer’s protocol using the above RNA library prep kit paired with the NEBNext Poly(A) mRNA Magnetic Isolation Module (New England Biolabs). Approximately 25 μL of RNA was diluted into 50 μL as input for poly(A) selected libraries. 16 cycles were used for the library amplification. Libraries were indexed using the NEBNext Multiplex Oligos for Illumina (New England Biolabs). The resulting library concentrations and size distributions for totalRNA, ribodepleted, and poly(A) selected RNA were measured by qPCR (Table 2) and an Agilent 2100 Bioanalyzer, respectively, as described above for the metagenomic libraries. Two equimolar pools were prepared on these measurements: one for totalRNA and one with ribodepleted and poly(A) selected RNA. The subsequent quality check on equimolar pools showed the absence of primer dimers for the totalRNA pool (Supplementary Figure 1A), but a significant amount of primer dimers in the ribodepleted and poly(A) selected pool (Supplementary Figure 1B–blue line). Therefore, an additional bead clean-up step using AMPure XP SPRI beads (Beckman Coulter, Villepinte, France) was used to remove primer dimers in that pool using a 0.7× dilution of beads according to the SPRIselect User guide (Supplementary Figure 1B–purple line). The totalRNA pool was sequenced in the same run as the metagenome pool. The ribodepleted and poly(A) selected pool was loaded on a separate V2-flow cell for a 2 × 250 paired-end sequencing run on an Illumina MiSeq.

### Bioinformatic Workflow for Processing of Sequencing Reads

Raw Illumina, paired-end metagenomic, and metatranscriptomic sequences were quality checked using FastQC v.0.11.8 (Andrews, 2014), and subsequently, quality filtered using the “illumina-utils” package of tools developed by Eren et al. (2013). All FastQC reports were compiled for easier inspection and visualization using MultiQC v.1.9 (Ewels et al., 2016). Once quality filtered processing of raw metatranscriptomic sequences for totalRNA, ribodepleted RNA, and poly(A) selected RNA were error corrected using SPAdes v3.11.1 (Bankevich et al., 2012). A co-assembly of preservation types was run *via* SqueezeMeta v1.3.0 (Tamames and Puente-Sánchez, 2019) utilizing megahit and the “–nobin” flag in order to halt further downstream processing.

For taxonomic inspection of the metagenome (DNA) and metatranscriptome (totalRNA) libraries, phyloFlash v3.4 (Gruber-Vodicka et al., 2020) was used. Briefly, PhyloFlash annotates short sequences from metagenome data to profile SSU rRNA to the SILVA (v138) rRNA gene database (Quast et al., 2013). We based our community analysis on extracted 16S and 18S genes as reference genomes for glacial algae are not available which makes taxonomic assignment *via* annotated genes difficult. The resulting tables of taxonomic units (NTUs) were imported into R and then converted into a phyloseq object. The data was then manipulated and visualized using the R packages dplyr v2.1.1 (Wickham et al., 2021), microbiome v1.14.0 (Lahti and Shetty, 2017), microViz v0.7.10 (Barnett et al., 2021), ggplot2 v3.3.5 (Wickham, 2016), phyloseq v1.36.0 (McMurdie and Holmes, 2013), and vegan v2.5.7 (Oksanen et al., 2016). The Generalized linear model – principal component analysis (GLM-PCA) package was used for the generation of the generalized principal component analysis (PCA) for non-normally distributed data (Townes et al., 2019).

## Results and Discussion

### General Trends From Snaefellsjökull and Langjökull Snow and Ice Samples

**FIGURE 2:**
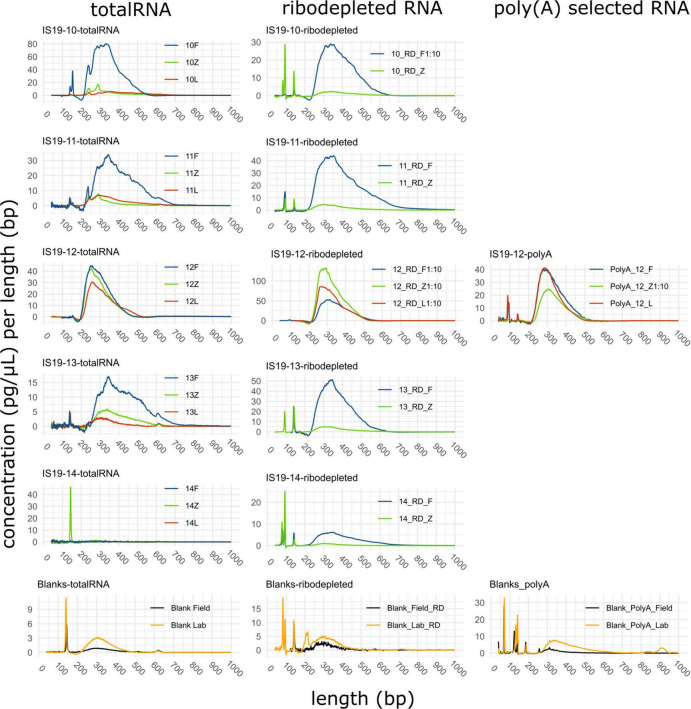
Length distributions of cDNA strands of single libraries from isolated RNA. Graphs show totalRNA, ribodepleted and poly(A) selected RNA in the left, middle, and right panel column, respectively. The preservation method is indicated in the legend as F, Z, and L for *Freeze*, *Zymo*, and *RNAlater*, respectively. All data was generated using a DNA high sensitivity assay on an Agilent Bioanalyzer 2100 system and generated data were analyzed and plotted with the R package “bioanalyzeR” (Foley, 2021). 10-fold dilution was used for those samples exceeding the optimal range of the DNA high sensitivity assay. Peaks < 150 bp correspond to primer-dimers generated during library preparation due to low input of RNA.

Our data show that all three preservation methods do preserve DNA and RNA from glacial samples. Samples from the high total organic carbon site Snaefellsjökull (IS19-10, 11, and 12) yielded higher DNA than the low total organic carbon site Langjökull (IS19-13 and 14) across both snow and ice samples, regardless of which preservation technique was used (Table 2). Furthermore, the two snow samples (IS19-10, and 13) were double and the snow-ice interface (IS19-12) sample yielded 20 times higher DNA concentrations than the two ice samples (IS19-11 and 14). One explanation for this could be that algal biomass in snow and interface samples is often much higher than in ice. This may be an artifact of how the samples were collected and how the biomass was distributed on/in the snow and ice matrices. For snow, we collected only the upper 3–5 mm of the snowpack–which was visually the darker snow and is most often associated with higher microbial and mineral particle loading (Lutz et al., 2015a; Cook et al., 2020; McCutcheon et al., 2021). For the ice samples, we had to collect the top 2–3 cm of the ice surface, which likely diluted the overall biomass in our samples because visual examination showed that minerals and pigmented microbes only covered the top 1–2 mm of the ice crystal surfaces, a phenomenon that has been reported previously on the Greenland Ice Sheet (Lutz et al., 2014; Cook et al., 2020). Among the three tested methods, RNAlater consistently led to lower yields. While all samples (except IS19-12) yielded RNA concentrations that were BDL as measured by the Qubit 2.0 fluorometer, libraries could nevertheless be successfully created (as demonstrated by the bioanalyzer traces, Figure 2). The successful amplification of RNA indicates an active microbial community in all our snow and ice samples at the time of sampling. In all cases, sample DNA averaged between 10 and 300 times higher than blank (FieldBL and LabBL) extraction DNA, while RNA library concentrations ranged between 1 and 12 times higher than the blank library concentrations.

Detectable concentrations of DNA were extracted successfully from all samples regardless of preservation type or biomass amount. Normalized DNA concentrations ranged from 0.7 ng/μl (FieldBL) to 590 ng/μl (12Z; Table 2) per 100 ml of filtered sample. The highest DNA concentrations were extracted from the *Zymo*-preserved samples (132.35 ± 256 ng/μl), followed by the *Freeze*-preserved samples (55.96 ± 77 ng/μl), while on average the *RNAlater*-preserved samples resulted in much lower average DNA yields (24.89 ± 42 ng/μl). In particular, the IS19-12 samples yielded average DNA concentrations that were up to 10× higher than in all other samples. Comparatively, DNA concentrations from snow and ice samples (when normalized to 100 ml sample fluid) from a site in the Canadian High Arctic ranged from BDL to 0.43 ng/μl (Trivedi et al., 2018). We attribute the high DNA concentration in IS19-12 to the visibly high amounts of biomass of glacial snow and ice algae within these samples (Figure 1C). This sample (IS19-12) also contained high TOC (Table 2; Winkel et al., 2022). The only sample to yield RNA concentrations above the detection limit of the Qubit was IS19-12. The highest RNA concentration was measured in the *Zymo*-preserved sample (12Z) (0.80 ng/μl).

The consistently lower DNA concentrations for *RNAlater*-preserved samples is likely a consequence of the additional washing steps used to remove excess salts from the RNAlater solution prior to extraction (as found by Saito et al., 2011), and the inhibition of ethanol-based extraction methods by high salt concentration solutions (Athanasio et al., 2016).

The potential loss of DNA and RNA during *RNAlater*-preserved sample washing is problematic for low-biomass glacial samples. In contrast to the use of RNAlater, many previous cryosphere studies addressing microbial community compositions froze filtered DNA samples at −20°C in the field (Larose et al., 2013; Christner et al., 2014; Maccario et al., 2014; Purcell et al., 2014; Lutz et al., 2015b,^2016^, ^2017^, 2019a,b; Bergk Pinto et al., 2019; Malard et al., 2019; Els et al., 2020; Ramoneda et al., 2020; Winkel et al., 2022), while few studies used *Zymo*-preservation (Trivedi et al., 2018, 2020; Matys et al., 2019; Dillon et al., 2020; Lumian et al., 2021), and even fewer used RNAlater plus freezing (Maccario et al., 2019). When no flash freezing and preservation of samples in a cryo-shipper exists, our results indicate that *Zymo*-preservation is a reasonable alternate option, although additional testing should be done to further help narrow the extraction boundary conditions for very low biomass samples as is often the case with snow and ice samples.

### Meta-Genomic/-Transcriptomic Library Preparation and Sequencing Yield

**FIGURE 3:**
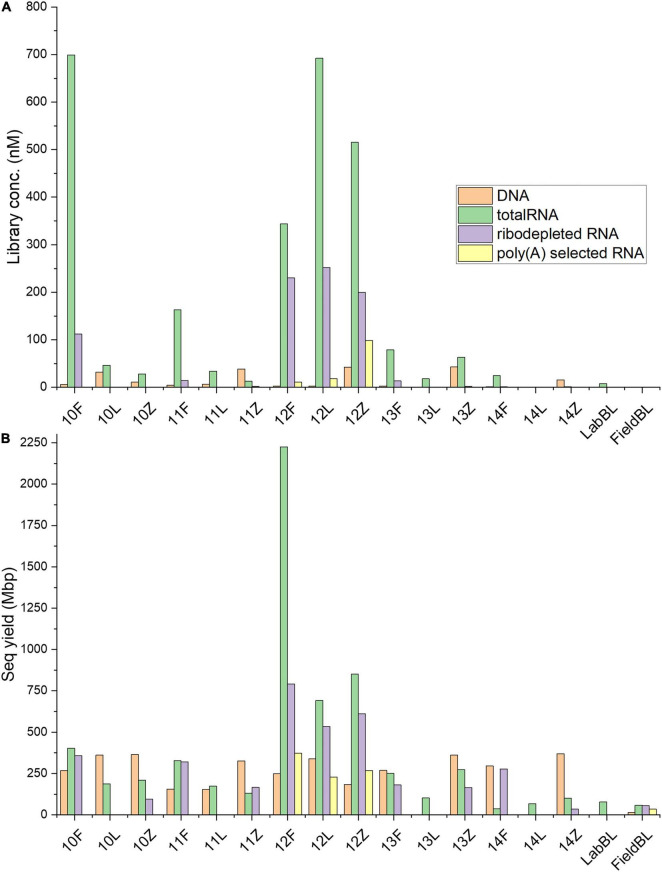
Library concentration and sequencing yield statistics. **(A)** Comparison between the concentrations (nM) of each prepared sample library as determined by qPCR. **(B)** Comparison between sequencing yields, in megabase pairs (Mbp) for each sample, clearly highlighting that both in terms of library concentrations and sequencing yields Site IS19-12 samples, regardless of treatment type, were clear outliers compared to the other samples (a notable exception was the totalRNA library concentration for sample 10F which was similarly high, yet in terms of sequencing yield site IS19-12 yielded, for all preservation types, the highest values–full details see Table 2).

Metagenomic libraries were prepared for all samples (labeled as “DNA” in Tables and Figures). Concentrations of metagenomic libraries ranged between BDL (13L and 14L) and 43.11 nM (13Z), with the averages for *Freeze*, *RNAlater*, and *Zymo* equaling 3.30 ± 1.85 nM, 8.18 ± 13.52 nM, and 30.16 ± 15.32 nM, respectively (Table 2 and Figure 3A). A one-way ANOSIM of these results showed that the only significant difference (*p* = 0.0468; Supplementary Table 1) was between *Freeze* and *Zymo*. Sequencing yield statistics (Table 2, Supplementary Table 2 and Figure 3B) were comparable across preservation types, ranging from <1 (14L) to 369 (14Z) megabasepairs (Mbp), and averages of 247 ± 54 Mbp (*Freeze*), 213 ± 169 Mbp (*RNAlater*), and 321 ± 79 Mbp (*Zymo*). Like the metagenomic library concentrations, the highest sequencing yields were obtained from *Zymo*-preserved samples (except for IS19-12). Our results show that sufficiently high-quality metagenomic libraries can be generated from low biomass environmental samples irrespective of the preservation method used.

TotalRNA library concentrations varied by almost three orders of magnitude (0.8 nM in 14L and 699.2 nM in sample 10F), with the averages for *Freeze*, *RNAlater*, and *Zymo* totaling 261.9 ± 272.73, 158.4 ± 299.06, and 124.44 ± 219.97 nM, respectively (Table 2 and Figure 2). The large standard deviations resulted from high RNA concentrations in sample 10F and the samples from site IS19-12 (Figure 3A). When comparing totalRNA library concentrations using a one-way ANOSIM, there was no significant difference between any of the preservation types (Supplementary Table 1). TotalRNA sequencing yields showed much larger ranges between samples (Table 2 and Figure 3B), with a low value of 36 Mbp (14F) and a high value of 2225 Mbp (12F). The average (Supplementary Table 2) across all samples was 402 ± 553 Mbp and for the preservation types: 648 ± 892 Mbp (*Freeze*), 244 ± 255 Mbp (*RNAlater*), and 313 ± 308 Mbp (*Zymo*). Note that the standard deviations are high due to the skewed values from site IS19-12 samples. When we remove IS19-12 samples, the average and standard deviation across all samples drops to 188 ± 110 Mbp (Supplementary Table 2).

The totalRNA sequencing yields show that the majority of samples had the highest yield from *Freeze*-preserved samples except for sites IS19-13 and IS19-14, where *Zymo*-preserved samples had the highest yields. Overall, *RNAlater*-preserved samples were lowest in sequencing yield, when compared to the other preservation methods except for site IS91-11 (Table 2). This seems to follow the trend of *RNAlater*-preserved samples likely having lower downstream statistics due to the additional upstream processing (rinsing filters before extraction). Unlike metagenomic data, totalRNA library concentration is positively correlated to totalRNA sequencing yield (Supplementary Figure 2). All totalRNA samples had undergone the same number of amplification cycles by PCR during library preparation. The measured library concentrations here can therefore be used as proxies for the initial extracted RNA from each sample. We back-calculated the amount of RNA used as starting material in the library preparation protocol which resulted in estimates 2 to 5 orders of magnitude lower than the recommended minimum of 5 ng of RNA (Supplementary Table 3). Although this approach might be less exact due to various biases in the actual duplication of DNA in PCR reactions, it allows us to approach the lower limits of RNA needed for the successful generation of metatranscriptomic data with currently available reagents.

The ribodepleted library concentrations ranged between 0.1 (10Z) and 252.0 (12L) nM (Table 2), with averages of 74.36 ± 97.80 nM (*Freeze*), 252 nM (*RNAlater*, *n* = 1), and 40.8 ± 88.72 nM (*Zymo*), respectively (Supplementary Table 2). Bioanalyzer traces (Figure 2) were used to assess quality before sequencing. Similarly, to the totalRNA library concentrations, samples from site IS19-12 as well as sample 10F greatly skewed the overall standard deviation, and while all ribodepleted samples for site IS19-12 were overwhelmingly the highest, they were all very similar in concentration (between 199.5 and 252 nM; Table 1 and Figure 3A). When excluding site IS19-12 samples and sample 10F, on average, ribodepleted library concentrations for *Freeze* samples were nine times higher than their *Zymo* counterparts. The yields of the ribodepleted samples ranged from 34 Mbp (14Z) to 792 Mbp (12F; Figure 3B), with an average (Supplementary Table 2) of 322 ± 236 Mbp, mostly due to the influence of site IS19-12 (as per RNA yield). When removed, the average and standard deviation both drop markedly (200 ± 112 Mbp). Overall, yields for *Freeze*-preserved samples were always greater than *Zymo*-preserved samples and in some cases as much as nine times higher (IS19-14, Table 2). However, when we compare preservation methods for samples from site IS19-12, the site with the highest DNA/RNA yields, all methods performed well, with *Freeze* (12F) yielding 792 Mbp, followed by *Zymo* (612 Mbp), and *RNAlater* (534 Mbp). This data shows that when considering a preservation method, the method seems less important when one deals with high biomass samples.

Libraries using poly(A) selection were only generated for site IS19-12 preserved samples, as our detailed microbial ecological analyses of this snow-ice interface sample (Winkel et al., 2022) revealed that the high biomass site was dominated by snow algae. Library concentrations ranged between 0.40 (FieldBL) and 98.4 (12Z) nM (Table 2 and Figure 3A). While the FieldBL and NEG samples had detectable RNA after library preparation, the concentrations are far lower than our real samples as also demonstrated in the bioanalyzer traces (Figure 2). The samples showed the same trend as noted in the totalRNA patterns (but opposite in the ribodepleted), where the *Zymo*-preserved sample yielded a much higher (98.4 nM) concentration than the *Freeze*- or *RNAlater*-preserved samples (which are both between 10 and 20 nM). Sequencing yield statistics for the five poly(A) selected samples [IS19-12 (F, L, and Z), FieldBL, and NEG; Figure 3B and Table 2] ranged from 34 Mbp (FieldBL) to 372 Mbp (12F) and had an average of 289 ± 74 Mbp when considering only site IS19-12. Sequencing yield followed the same trend as that of ribodepleted yields, where *Freeze*-preserved was highest, followed by *Zymo* and *RNAlater*, respectively. It should be noted that while poly(A) selected library concentrations were much lower than those in the ribodepleted samples for IS19-12, the yield was only about half. This points to the quality of the normalization of sample libraries prior to sequencing. It should also be noted that while controls were sequenced along with the variably preserved sample types, any sequencing yield in the FieldBL and LabBL blanks could be a signal of carryover from the samples with higher biomass (e.g., site IS19-12).

We obtained comparable sequence yields from most samples, which indicates that enough nucleic acids were extracted to assure successful library preparation and sequencing (for both DNA and RNA). However, the lack of metagenomic sequences in samples 13L and 14L suggests the co-extraction of inhibitory substances in these samples, because all showed similar DNA concentrations after extraction. This seems plausible, as polymerase chain reactions used to amplify the later sequenced parts of DNA during library preparation, can be inhibited by co-extracted molecules (Kreader, 1996; Schrader et al., 2012), which was also found to be the case in samples from snow and ice environments (Takeuchi, 2002). Additionally, we note that in low TOC samples (IS19-13 and −14) *RNAlater* performance was less than that of *Zymo* or *Freeze* preservation, suggesting the use of *Zymo*-preservation on samples where TOC% may be low and chemical preservation is used.

The number of sequences generated from the metatranscriptomes (totalRNA, ribodepleted, and poly(A) selected) suggests good performance of the chosen protocols, which is surprising given the initial very low amounts of RNA extracted from the samples. Although the presented sequence yields cannot be connected directly to the quality of the used preservation methods, data show that we can generate sequences with much lower amounts of RNA than recommended in standard protocols developed on easier to handle sample material. However, as we also generated sequences in our blank samples, we analyzed the taxonomic composition of our samples and blanks in order to evaluate which samples resulted in biological meaningful datasets.

### Taxonomic Classification of DNA and TotalRNA

**FIGURE 4:**
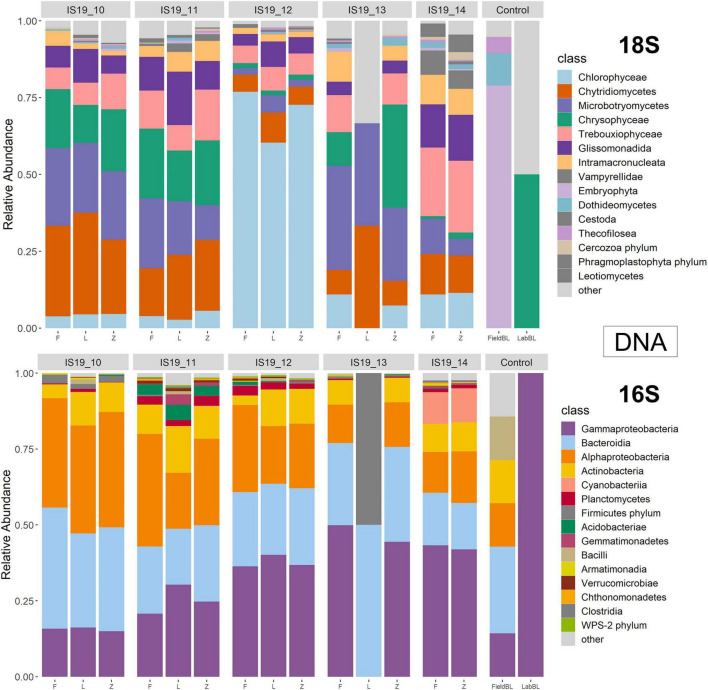
Relative abundance annotated rRNA reads from DNA (metagenome) sample separated by 18S and 16S. Reads were processed through phyloFlash using the SILVA 138 database. Barplots are scaled to relative abundance and taxonomic classification is shown on the class level.

**FIGURE 5:**
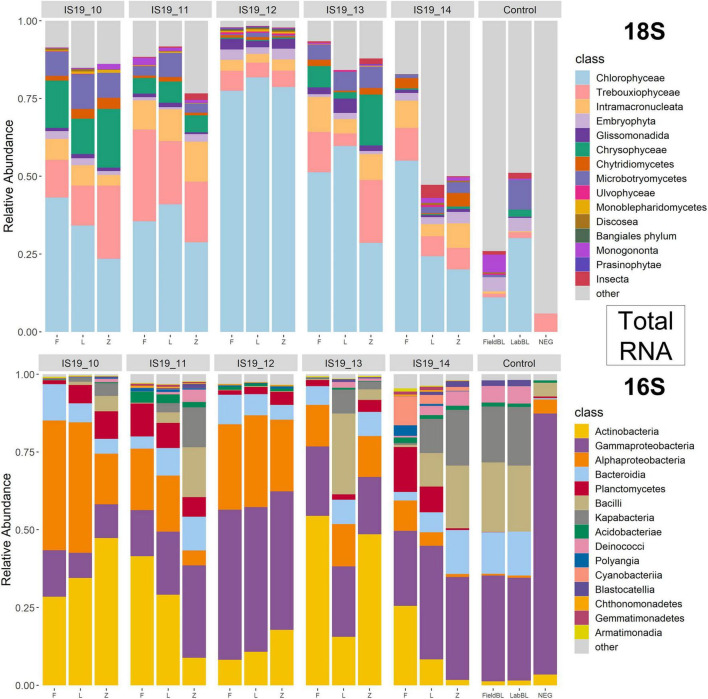
Relative abundance annotated rRNA reads from totalRNA (metatranscriptome) sample separated by 18S and 16S. Reads were processed through phyloFlash using the SILVA 138 database. Barplots are scaled to relative abundance and taxonomic classification is shown on the class level.

We used small subunit (SSU) rRNA genes recovered from the metagenome and metatranscriptomic sequences to evaluate the taxonomic diversity in each sample in relation to preservation type, organic carbon content (i.e., TOC), and sequencing library concentrations. Relative abundance bar plots of all samples (including control samples) on the class level reveal diverse communities within the samples as determined by the presence of recovered SSU rRNA genes (Figures 4, 5). The DNA barplots are represented by a total of 25,298 SSU rRNA sequences (average/sample: 1,413 sequences, Supplementary Table 4; SSU sequences were not recovered for IS19-14L so this sample was removed), while the totalRNA relative abundance is represented by 4,663,984 sequences (average/sample: 259,110 sequences; Supplementary Table 4). The reason for this large discrepancy is because when sequencing all RNA in a sample, over 90% is often represented by ribosomal RNA (Shakya et al., 2019), whereas with shotgun metagenomic sequencing, the proportion of rRNA genes is much lower. When our *Freeze* data is compared against amplicon data from Winkel et al. (2022) for the same samples (that were also sequenced from *Freeze*-preserved samples), our evaluation yielded similar taxonomic classifications for both 16S and 18S rRNA gene sequencing, further increasing our confidence that recovered rRNA (from both metagenomes and metatranscriptomes) was classified correctly. Organisms that we expected to find in these samples based on the amplicon data include (i) from the 16S data: *Actinobacteria, Bacteriodia, Alphaproteobacteria*, and *Gammaproteobacteria*, and (ii) from the 18S data: *Chlorophyceae, Chryosophyceae, Trebouxiophyceae*, and *Phragmoplastophya*, all of which are key algae in glacial ecosystems and which have been documented before on the same glaciers in Iceland (Lutz et al., 2015a,^2018^; Winkel et al., 2022).

**FIGURE 6:**
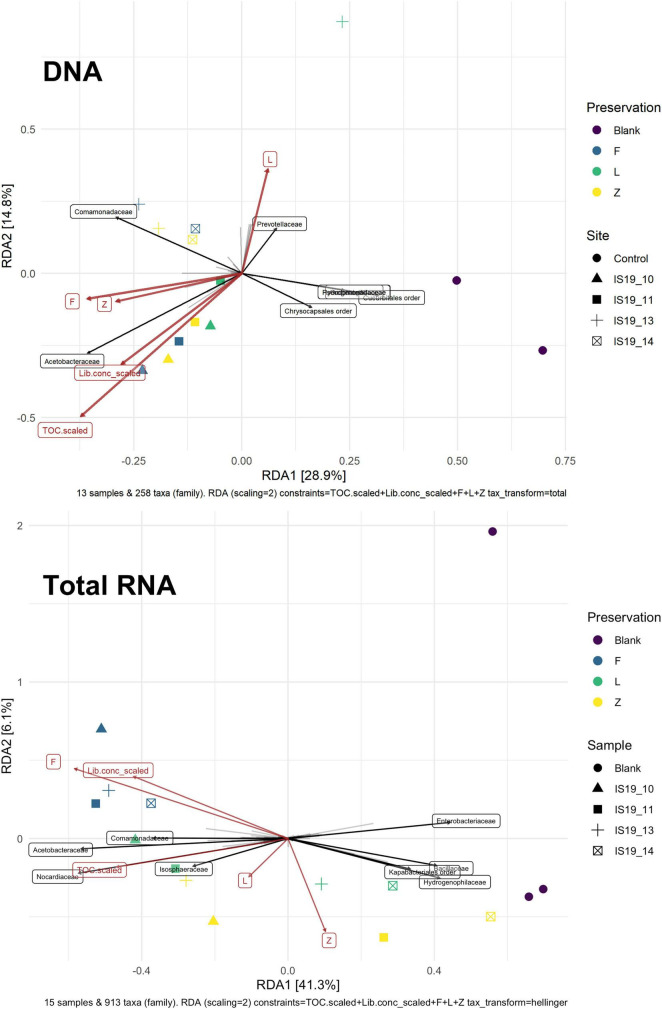
Constrained ordination of count tables of annotated taxa (excluding site IS19-12) with preservation methods, normalized TOC, DNA, and totalRNA library concentrations as explanatory variables. Light gray vectors indicate taxa loading, where the relative length indicates a given concentration. Those taxa with the highest contribution are shows as black vectors and labeled with the taxa. For DNA the taxonomic signatures show a clear difference between samples and blanks which is not the case for totalRNA where samples with low amounts of extracted RNA are placed near blanks. For DNA, explanatory variables are all correlated with taxonomy of the samples, while for totalRNA library concentration, TOC and Freeze are least correlated with blank samples while Zymo and RNAlater correlate with samples and tend to cluster with blanks. Note that sample IS19-14L is not included in the DNA RDA plot as it was removed due to low sequence count.

**FIGURE 7:**
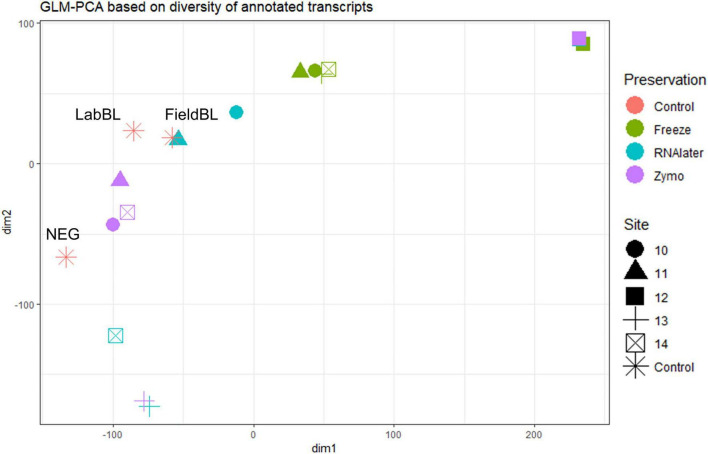
GLM-PCA plot of totalRNA by site and by preservation type based on annotated genes from the preservation type coassemblies. Most interesting is that all site IS19-12 samples cluster together, regardless of preservation type (full squares in upper right corner). Additionally, the *Freeze*-preserved samples also cluster together (green colored symbols), while there appears to be some overlap between *Zymo*- and *RNAlater*-preserved samples with control samples.

We found that preservation type had less of an effect on community composition compared to a metric such as successful sequencing library preparation (as evidenced by the variations in library concentration; Figure 3). Samples with low (or BDL) library concentrations (e.g., DNA: 13L, RNA: 11Z, 13L, 14L, and 14Z) resembled the control samples (FieldBL, LabBL, or NEG). To explore this further we ran a constrained ordination (with site IS19-12 removed as it is very different as snow algae dominated and this highly biases the signal of the other samples, Figure 6) with preservation type, library concentration, and TOC. For both DNA and totalRNA we found that successful library preparation was not correlated to the taxonomic composition of the blank samples, as denoted by the library concentration vectors in the constrained ordination. Additionally, our DNA data (Figure 3 and Table 2) suggest that almost all *Zymo*-preserved samples yielded the highest library concentrations, independent of the amount of filtered sample. We ran an additional principle component analysis (for non-normally distributed data, GLM-PCA) to assess the ordination of annotated transcripts from the co-assembly of all totalRNA samples (Figure 7), which reveals that all *Freeze*-preserved samples, clustered together suggesting high similarity of annotated transcripts and therefore similar library preparation (Figure 2). This further indicates that sample volume is the most important factor when collecting glacial snow and ice and not preservation type. Because these samples clustered so well together it tells us that preservation type is not biasing our recovery of transcripts in sample IS19-12.

## Recommendations and Conclusion

Our results reveal that the different methods of nucleic acid preservation from glacial snow and ice samples invariably affects the yield and quality of DNA and RNA recovered. We showed that preservation by freezing in liquid nitrogen (*Freeze*) and chemically preserving with *Zymo* (DNA/RNA Shield as part of the ZymoBIOMICS DNA/RNA Miniprep kit) result in comparable preservation efficiency, while samples chemically preserved with *RNAlater* appeared to lead to poorer preservation efficacy. DNA yield was strongly influenced by the biomass content of the sample, and the amount of sample volume filtered noticeably impacted extraction concentration and therefore the quality of the library preparation. The biomass contents are invariably linked to the amount of sample volume filtered and in turn to the density of microbial particulates in snow or ice samples. The RNA yield from samples was strongly affected by the total volume of sample filtered, and many of our low biomass samples yielded RNA concentrations that were too low for successful library preparation. Taxonomy based on SSU rRNA gene presence indicated similar profiles between DNA and totalRNA libraries and showed no selective differences between preservation types. We observed that the apparent taxonomic diversity was sensitive to the amount of sample filtered. Metatranscriptomes of samples for which low volumes were filtered (e.g., 100 ml) were difficult to evaluate as they revealed signals similar to the control blanks. Nevertheless, we could show that a successful generation of metatranscriptomic data from snow and ice is possible with much lower amounts of RNA as starting material than in the manufacturer’s recommendations. However, our data show that below a starting material of ∼1 pg the taxonomic signature started to show taxonomic classes only found in the blanks. This is likely due to the amplification of contaminant DNA present in library prep kits or used labware, which does not become apparent when sufficient RNA/DNA is supplied. We found that flash-freezing and Zymo-preservation are comparable in preservation efficacy, and favorable to RNAlater–which is especially disadvantageous in low biomass environments. We also found that DNA and RNA yield and quality are strongly dependent on (filtered) sample volume, and we, therefore, recommend maximizing filtration throughput during sample processing.

Effective preservation of short-lived nucleic acid such as RNA from biological samples is paramount in many field settings. We, therefore, suggest that in a typical field-sampling scenario when snow or ice containing variable microbial biomass proportions is the research target, three things should be taken into consideration: (i) Is the sample low (ice) or high (snow) biomass? (ii) How much material can be collected based on the sample type? and (iii) Does one have the ability to collect replicates? We argue based on our findings presented above that at a minimum, 200 ml of melted ice or snow should be collected in order to capture a representative environmental RNA signal. Additionally, if criteria one suggests that the samples are most likely low in overall biomass content, a much larger volume (>200 ml) should be filtered; if that is not possible (i.e., due to possible clogging of the filter due to particulates), then replicate filters should be collected for pooling after nucleic acid extraction. Finally, field and lab blanks should also be taken for taxonomic comparison. In amplicon-based studies, where low biomass samples are collected, samples often taxonomically resemble kit or reagent contaminants which can cause downstream issues with interpretation (Salter et al., 2014; Karstens et al., 2019). Additionally, we feel that these recommendations and suggestions can be applied to other environments and ecosystems where on-site sample preservation is important, such as in difficult to access environments, during long-term sampling or monitoring campaigns, and for low biomass samples.

## Data Availability Statement

The datasets presented in this study can be found in online repositories. The names of the repository/repositories and accession number(s) can be found below: https://www.ncbi.nlm.nih.gov/, SAMN26570417–SAMN26570460.

## Author Contributions

LGB, CBT, JAB, and RM led the design of the study and conducted the fieldwork. CBT, CL, CK, and MW carried out the laboratory work. CBT, CK, and DVR performed the bioinformatic analysis. CBT wrote the first draft of the manuscript. All authors contributed guidance and edits to the final manuscript preparation.

## Conflict of Interest

The authors declare that the research was conducted in the absence of any commercial or financial relationships that could be construed as a potential conflict of interest.

## Publisher’s Note

All claims expressed in this article are solely those of the authors and do not necessarily represent those of their affiliated organizations, or those of the publisher, the editors and the reviewers. Any product that may be evaluated in this article, or claim that may be made by its manufacturer, is not guaranteed or endorsed by the publisher.
