# Supplementary material for: DNA/RNA Preservation in Glacial Snow and Ice Samples
Source: Front Microbiol. 2022 May 23;13:894893. doi: 10.3389/fmicb.2022.894893 (PMC9168539; doi:10.3389/fmicb.2022.894893)
Supplement: Supplementary file 1 [file Table_1.docx]

***Supplementary Material***


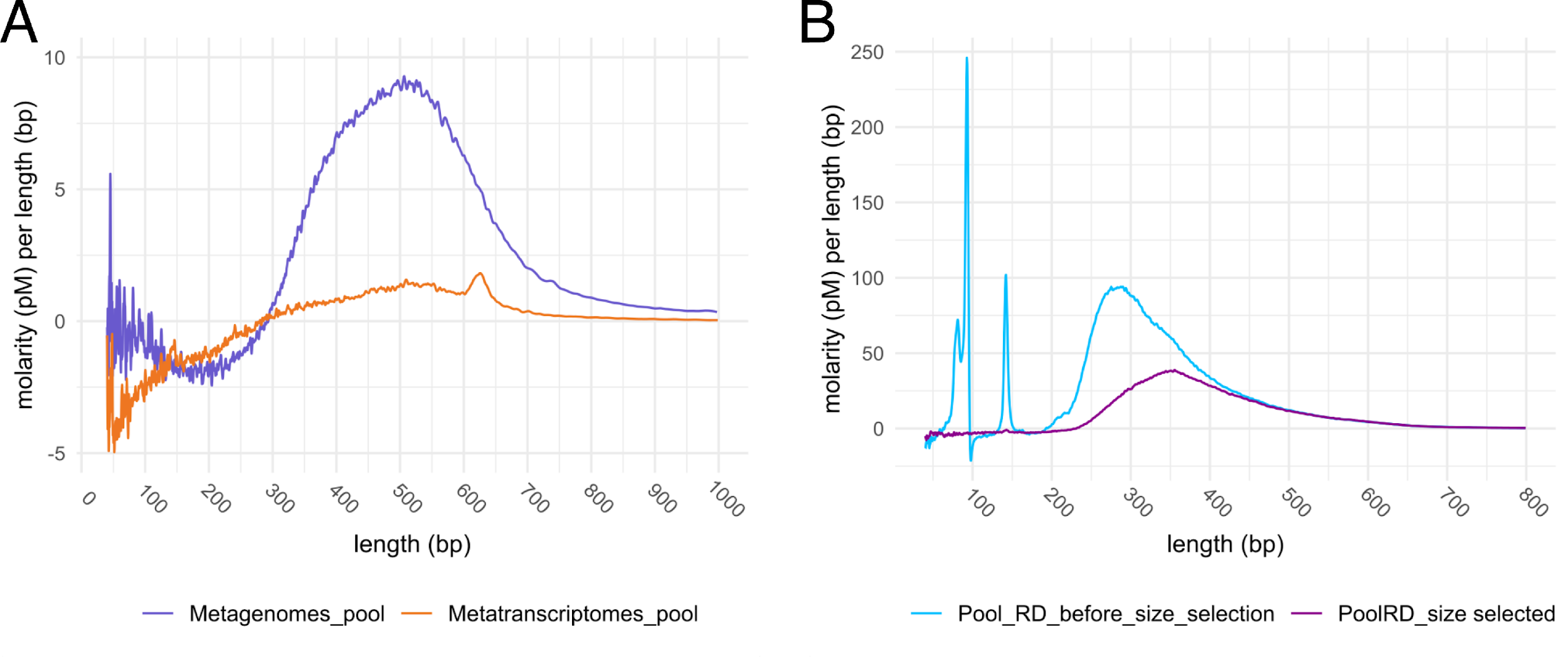


**SI Figure 1. Length distributions of DNA and cDNA strands of library pools before sequencing.** A) Metagenomic and metatranscriptomic libraries prepared from all pooled samples for total DNA (purple pattern) and RNA (orange pattern), respectively. Size distribution shows the recommended values according to Illumina recommendations (~300 to 800 bp). B) The metatranscriptomic pool of all ribodepleted and poly(A) selected RNA libraries. The peaks < 150 bp (blue pattern) correspond to primer-dimers generated during library preparation due to low input of cDNA. An additional size selection step before sequencing excluded these peaks (purple pattern) and assured sequencing of cDNA originating from sampled organisms rather than artefacts from library preparation. Pools were analyzed using a DNA high sensitivity assay on an Agilent Bioanalyzer 2100 system and generated data were analyzed and plotted with the R package “bioanalyzeR” (Foley, 2021).


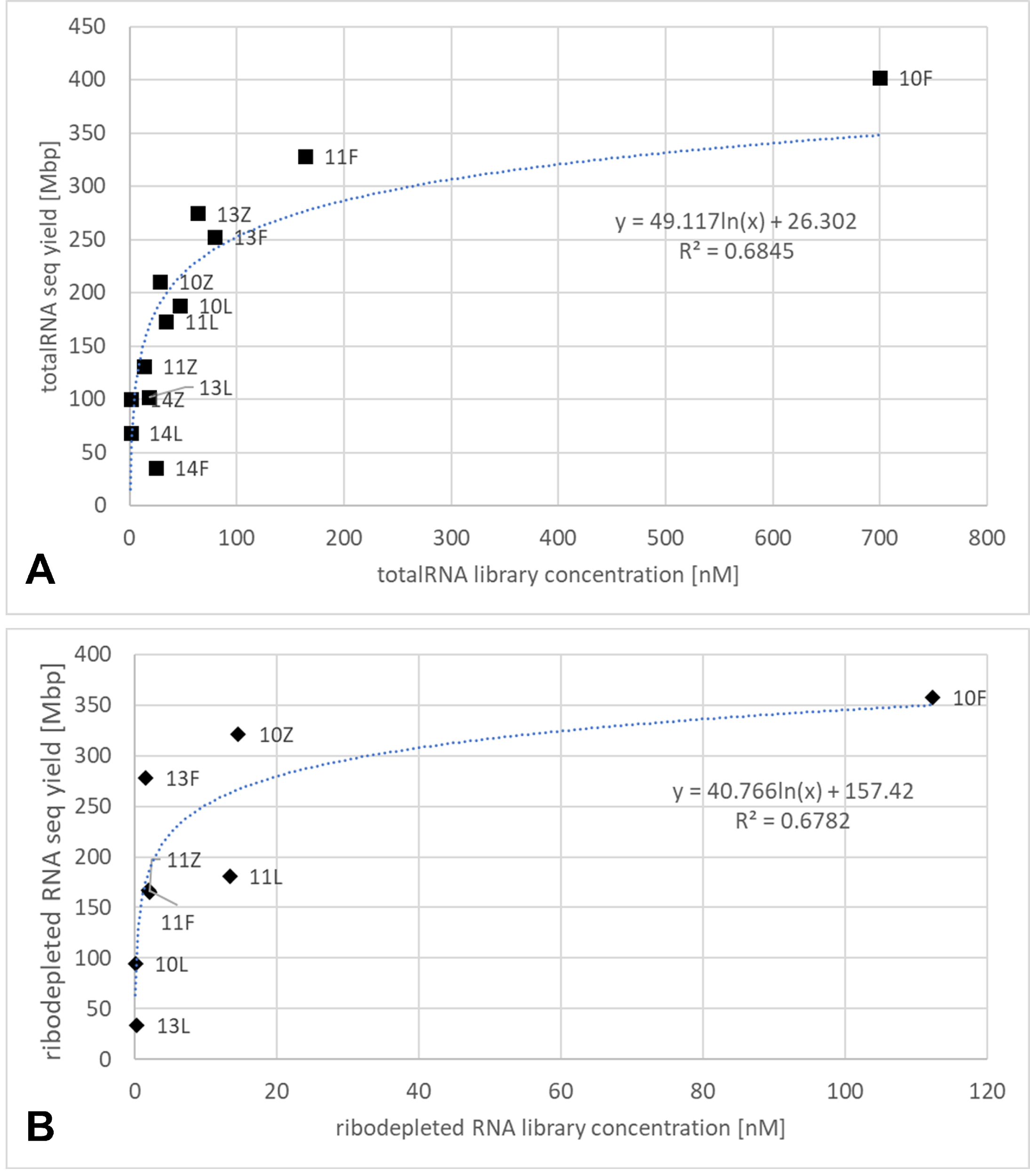


**SI Figure 2. Correlation plots comparing totalRNA and ribodepleted RNA library concentrations versus sequencing yield (with site IS19-12 samples removed because of bias due to much higher biomass than all other samples).** A) Correlation plot showing a logarithmic trend between the totalRNA concentration (nM) of the sample library preparations and the associated sequencing yield (Mbp). Complementing this, is the plot in (B) also showing a logarithmic trend between the concentration of the ribodepleted RNA libraries and the sequencing yield. These trends strengthen the hypothesis that successful library preparation is more important than preservation type.

**SI Table 1. One-way ANOSIM results.** One-way ANOSIM tests helped determine significant differences between preservation types across library concentration, and sequencing yield. For any tests showing significance, a Bonferonni correction was applied. Only one comparison was significant: that between Freeze and Zymo metagenome library concentrations (p=0.0468, highlighted in yellow), all other comparisons were not significant.

| **DNA extraction concentration** | | | R=0.04209 | | **DNA library concentration** | | | R=0.3266* | | **DNA seq yield** | |  | | R=0.2952* | | |
| --- | --- | --- | --- | --- | --- | --- | --- | --- | --- | --- | --- | --- | --- | --- | --- | --- |
|  | Freeze | RNA later | Zymo | Control |  | Freeze | RNA later | Zymo | Control |  | Freeze | RNA later | Zymo | | Control |  |
| Freeze |  |  |  |  | Freeze |  |  |  |  | Freeze |  |  |  | |  |  |
| RNA later | 0.1876 |  |  |  | RNA later | 1 |  |  |  | RNA later | 1 |  |  | |  |  |
| Zymo | 0.6353 | 0.2145 |  |  | Zymo | **0.0468** | 0.3246 |  |  | Zymo | 0.3804 | 1 |  | |  |  |
| Control | 0.3386 | 0.434 | 0.1895 |  | Control | 0.5862 | 1 | 0.3006 |  | Control | 0.2892 | 1 | 0.2958 | |  |  |
| **RNA extraction concentration** | | | R=-0.09124 | | **totalRNA library concentration** | | | R=-.08909 | | **totalRNA seq yield** | |  | | R=-0.02919 | | |
|  | Freeze | RNA later | Zymo | Control |  | Freeze | RNA later | Zymo | Control |  | Freeze | RNA later | Zymo | | Control |  |
| Freeze |  |  |  |  | Freeze |  |  |  |  | Freeze |  |  |  | |  |  |
| RNA later | 1 |  |  |  | RNA later | 0.508 |  |  |  | RNA later | 0.2656 |  |  | |  |  |
| Zymo | 1 | 1 |  |  | Zymo | 0.4401 | 0.9133 |  |  | Zymo | 0.4694 | 0.8182 |  | |  |  |
| Control | 1 | 1 | 1 |  | Control | 0.4666 | 0.5285 | 0.7182 |  | Control | 0.5164 | 0.43 | 0.3876 | |  |  |
|  |  |  |  |  | **ribodepleted library concentration** | | | R=0.03656 | | **ribodepleted seq yield** | | | | R=-0.005223 | | |
|  | * = Bonferroni corrected | | |  |  | Freeze | RNA later | Zymo | Control |  | Freeze | RNA later | Zymo | | Control |  |
|  |  |  |  |  | Freeze |  |  |  |  | Freeze |  |  |  | |  |  |
|  |  |  |  |  | RNA later | 0.1851 |  |  |  | RNA later | 0.2697 |  |  | |  |  |
|  |  |  |  |  | Zymo | 0.2724 | 0.0813 |  |  | Zymo | 0.4601 | 0.8226 |  | |  |  |
|  |  |  |  |  | Control | 0.4807 | 0.5226 | 0.5666 |  | Control | 0.4836 | 0.3821 | 0.3801 | |  |  |
|  |  |  |  |  | **poly(A) library concentration** | | | R=-0.0298 | | **poly(A) seq yield** | |  | R=-0.03963 | | | |
|  |  |  |  |  |  | Freeze | RNA later | Zymo | Control |  | Freeze | RNA later | Zymo | | Control |  |
|  |  |  |  |  | Freeze |  |  |  |  | Freeze |  |  |  | |  |  |
|  |  |  |  |  | RNA later | 1 |  |  |  | RNA later | 1 |  |  | |  |  |
|  |  |  |  |  | Zymo | 1 | 1 |  |  | Zymo | 1 | 1 |  | |  |  |
|  |  |  |  |  | Control | 0.5269 | 0.5292 | 0.5227 |  | Control | 0.5357 | 0.5269 | 0.531 | |  |  |

**SI Table 2. Library concentration [nM] and sequencing yield [Mbp] averages and standard deviations.** Statistics were calculated to evaluate variability or trends between the different preservation types (excluding control samples). Additionally, in order to evaluate how the total average and standard deviations in the samples changed – in a second evaluation sample IS19-12 was removed from this assessment (Avg w/o site IS19-12) to evaluate if and how the high biomass of this sample affected the statistics. Below the new average where IS19-12 was removed the % Change (bolded) indicates a positive (increase) or negative (decrease) percent change when site IS19-12 was removed as compared to the initial average (Avg ± Stdev). For metagenome libraries and yields, this had little impact, while a much larger impact was seen for the averages in totalRNA and ribodepleted libraries and sequencing yields.

|  | **DNA** | **totalRNA** | **ribodepleted RNA** | **poly(A) RNA** | **DNA yield** | **totalRNA yield** | **ribodepleted yield** | **poly(A) yield** |
| --- | --- | --- | --- | --- | --- | --- | --- | --- |
| Avg ± Stdev | 13.88 ± 16.33 | 181.58 ± 253.56 | 75.25 ± 103.4 | 42.5 ± 48.55 | 264 ± 108 | 402 ± 553 | 322 ± 236 | 289 ± 74 |
| Freeze | 3.3 ± 1.85 | 261.9 ± 272.73 | 74.36 ± 97.8 | 10.9 | 247 ± 54 | 648 ± 892 | 386 ± 236 | 372 |
| RNAlater | 8.18 ± 13.52 | 158.4 ± 299.06 | 252 | 18.2 | 213 ± 169 | 244 ± 255 | 534 | 229 |
| Zymo | 30.16 ± 15.32 | 124.44 ± 219.97 | 40.8 ± 88.72 | 98.4 | 321 ± 79 | 313 ± 308 | 214 ± 229 | 267 |
|  |  |  |  |  |  |  |  |  |
| Avg w/o site IS19-12 | 13.43 ± 15.57 | 97.65 ± 194.61 | 18.29 ± 38.43 | - | 266 ± 117 | 188 ± 110 | 200 ± 112 | - |
| **% Change** | **-7.69** | **-85.71** | **-358.33** | **-** | **0.38** | **-113.83** | **-77.44** | **-** |

**SI Table 3: Estimation of initial amounts of RNA used for library preparation based on measurements of final libraries and amplifications used in the library preparation protocol.** Average size of libraries are results from Bioanalyzer measurements between 200 and 2000 bp (excluding primer dimers). The recommended amount RNA as starting material in the protocol of used NEBNext Ultra II Direction RNA Library Prep kit ranges between 5 ng – 1 µg of totalRNA.

| Sample | Molarity of library measured by qPCR  [nM] | Average size of library [bp] | Conc. of RNA libraries inferred from molarity and average size  [ng/µL] | Conc. of RNA in original sample based on 16 cycles of PCR  [pg/µL] | RNA input in library prep  [ng] |
| --- | --- | --- | --- | --- | --- |
| 10Z | 28.2 | 471 | 8.65 | 0.132 | 1.58E-03 |
| 11Z | 13.3 | 446 | 3.87 | 0.059 | 7.08E-04 |
| 12Z | 515.7 | 334 | 111.97 | 1.708 | 2.05E-02 |
| 13Z | 63.6 | 408 | 16.86 | 0.257 | 3.09E-03 |
| 14Z | 1.4 | 315 | 0.28 | 0.004 | 5.19E-05 |
| Field BL | 0.8 | 381 | 0.21 | 0.003 | 3.82E-05 |
| 10L | 46.6 | 504 | 15.26 | 0.233 | 2.79E-03 |
| 11L | 34.0 | 461 | 10.20 | 0.156 | 1.87E-03 |
| 12L | 692.5 | 358 | 161.15 | 2.459 | 2.95E-02 |
| 13L | 18.1 | 365 | 4.28 | 0.065 | 7.85E-04 |
| 14L | 0.8 | 409 | 0.20 | 0.003 | 3.73E-05 |
| Lab BL | 8.0 | 338 | 1.75 | 0.027 | 3.21E-04 |
| 10F | 699.2 | 346 | 157.24 | 2.399 | 2.88E-02 |
| 11F | 163.3 | 415 | 44.05 | 0.672 | 8.07E-03 |
| 12F | 343.7 | 320 | 71.49 | 1.091 | 1.31E-02 |
| 13F | 78.8 | 443 | 22.70 | 0.346 | 4.16E-03 |
| 14F | 24.5 | 481 | 7.67 | 0.117 | 1.40E-03 |
| NEG | 0.7 | 462 | 0.21 | 0.003 | 3.88E-05 |

**SI Table 4. SSU rRNA sequence counts used for the taxonomic classification barplots and the ordination analyses.** Counts represent those of only SSU rRNA gene sequences after processing with phyloFlash. Averages calculated at the bottom of the table are used to highlight the large bias site IS19-12 has on count values. Shown is the average of all counts with and without site IS19-12.

| **Sample** | **DNA seq count** | **totalRNA seq count** |
| --- | --- | --- |
| IS19_10F | 1578 | 294042 |
| IS19_10L | 2496 | 151427 |
| IS19_10Z | 2468 | 71643 |
| IS19_11F | 928 | 370961 |
| IS19_11L | 620 | 189215 |
| IS19_11Z | 1658 | 74474 |
| IS19_12F | 2002 | 1953078 |
| IS19_12L | 2712 | 481554 |
| IS19_12Z | 1422 | 650015 |
| IS19_13F | 1662 | 192438 |
| IS19_13L | 10 | 11950 |
| IS19_13Z | 2224 | 34138 |
| IS19_14F | 1152 | 37166 |
| IS19_14L | 0 | 16422 |
| IS19_14Z | 1604 | 58588 |
| IS19_FieldBL | 66 | 48087 |
| IS19_LabBL | 8 | 27942 |
|  |  |  |
| Average ALL | 1413 | 259110 |
| Average w/o IS19-12 | 1177 | 112750 |

**SI Table 5. SSU rRNA sequencing counts from TotalRNA (metatranscriptome) sample libraries.** These tables were generated using phyloFlash for all sample sites and controls, and include full taxonomic assignments and associated sequencing counts. This table is provided as a Microsoft Excel file (data sheet 1).

**SI Table 6. SSU rRNA sequencing counts from DNA (metagenome) sample libraries.** These tables were generated using phyloFlash for all sample sites and controls, and include full taxonomic assignments and associated sequencing counts. This table is provided as a Microsoft Excel file (data sheet 2).

**References**

Foley, J. (2021). *bioanalyzerR: R tools for Agilent electrophoresis data*. Available at: https://github.com/jwfoley/bioanalyzeR.
